# HIGH AROUSAL AFFECT RELATED TO WORSE WORKING MEMORY BUT MORE FOR YOUNGER ADULTS

**DOI:** 10.1093/geroni/igae098.2706

**Published:** 2024-12-31

**Authors:** Julie Kircher, Eric Cerino, Jacqueline Mogle, Susan Charles

**Affiliations:** The Pennsylvania State University, State College, Pennsylvania, United States; Northern Arizona University, Flagstaff, Arizona, United States; Clemson University, Clemson, South Carolina, United States; University of California Irvine, Irvine, California, United States

## Abstract

Studies have found that high emotional arousal states, assessed by reports of negative affect, are often related to poorer working memory. Findings for positive affect (e.g., excitement or joyful), however, are more mixed, with some studies indicating that higher arousal is linked to better cognitive task performance. The purpose of this study was to examine how reports of negative and positive affect throughout the day are associated with verbal working memory, and how this relationship may vary by age. We hypothesized that moments of high negative or positive affect will be related to poorer cognitive task performance, and that age would moderate the relationship between affect and cognition. Data from the Effects of Stress on Cognitive Aging, Physiology, and Emotion (ESCAPE) study consisted of five assessments throughout the day across 14 days among 263 community residents ranging from 25-65 years old (Mage = 46.5). At each assessment, participants completed the n-back adapted for mobile phones and reported the current affect. Across all ages, higher levels of reported negative and positive affect at each assessment were related to worse momentary working memory (γNA = -.0004, SE =.0002, p =.02; γPA = -0.0003, SE =.0002, p =.04) and younger age was related to worse working memory as levels of positive affect increased.

